# Seasonal Changes in the Plant Growth-Inhibitory Effects of Rosemary Leaves on Lettuce Seedlings

**DOI:** 10.3390/plants11050673

**Published:** 2022-03-01

**Authors:** Kwame Sarpong Appiah, Richard Ansong Omari, Siaw Onwona-Agyeman, Christiana Adukwei Amoatey, John Ofosu-Anim, Abderrazak Smaoui, Abdelkarim Ben Arfa, Yoko Suzuki, Yosei Oikawa, Shin Okazaki, Keisuke Katsura, Hiroko Isoda, Kiyokazu Kawada, Yoshiharu Fujii

**Affiliations:** 1 Department of International Innovative Agricultural Science, Tokyo University of Agriculture and Technology, 3-5-8 Saiwaicho, Fuchu 183-8509, Tokyo, Japan; yosei@cc.tuat.ac.jp (Y.O.); sokazaki@cc.tuat.ac.jp (S.O.); kkatsura@go.tuat.ac.jp (K.K.); yfujii@cc.tuat.ac.jp (Y.F.); 2 Department of Crop Science, College of Basic and Applied Science, University of Ghana, Legon, Accra P.O. Box LG 44, Ghana; camoatey@ug.edu.gh; 3 Leibniz Centre for Agricultural Landscape Research, Institute of Land Use Systems, Eberswalder Str. 84, 15374 Muencheberg, Germany; talk2jafakingonline@gmail.com; 4 Institute of Agriculture and Horticulture, Faculty of Life Science, Humboldt-University of Berlin, Albrecht-Thaer-Weg 5, 14195 Berlin, Germany; 5 Institute of Agriculture, Tokyo University of Agriculture and Technology, 3-5-8 Saiwaicho, Fuchu 183-8509, Tokyo, Japan; agyeman@cc.tuat.ac.jp; 6 School of Architecture and Science, Central University, Tema P.O. Box 2305, Ghana; jofosuanim@gmail.com; 7 Centre of Biotechnology of Borj Cédria, BP 901 Hammam-Lif, Borj Cedria 2025, Tunisia; abderrazak.smaoui@gmail.com; 8 L’Institut des Régions Arides, Route du Djorf Km 22.5, Médenine 4119, Tunisia; abdelkarim.benarfa@ira.rnrt.tn; 9 Aromatic Repos, AHOLA, A2 Soleil Jiyugaoka, 1-21-3, Jiyugaoka, Meguro 152-0035, Tokyo, Japan; yoko86252539@gmail.com; 10 School of Life and Environmental Sciences, University of Tsukuba, Tennoudai 1-1-1, Tsukuba 305-8572, Ibaraki, Japan; isoda.hiroko.ga@u.tsukuba.ac.jp

**Keywords:** Mediterranean climate, elongation, allelochemicals, specific activity, phytotoxicity

## Abstract

Plant biodiversity has been studied to explore allelopathic species for the sustainable management of weeds to reduce the reliance on synthetic herbicides. Rosemary (*Rosmarinus officinalis* L., syn *Salvia rosmarinus* Spenn.), was found to have plant growth-inhibitory effects, and carnosic acid was reported as an allelochemical in the plant. In this study, the effects of seasonal variation (2011–2012) on the carnosic acid concentration and phytotoxicity of rosemary leaves from two locations in Tunisia (Fahs and Matmata) were investigated. The carnosic acid concentration in rosemary leaves was determined by HPLC, and lettuce (*Lactuca sativa* L.) was used as the receptor plant in the phytotoxicity bioassay. The highest carnosic acid concentration was found in rosemary samples collected in June 2011, which also had the highest inhibitory activity. Furthermore, a significant inverse correlation (*r* = −0.529; *p* < 0.01) was found between the inhibitory activity on lettuce hypocotyl and the carnosic acid concentration in rosemary leaves. Both temperature and elevation had a significant positive correlation with carnosic acid concentration, while rainfall showed a negative correlation. The results showed that the inhibitory effects of rosemary leaf samples collected in summer was highest due to their high carnosic acid concentration. The phytotoxicity of rosemary needs to be studied over time to determine if it varies by season under field conditions.

## 1. Introduction

Interference from weeds can have a significant impact on the growth and development of field crops, resulting in substantial crop production losses [1]. The use of synthetic herbicides to minimize crop loss due to weed infestation has become the predominant weed control strategy. However, the global increase in herbicide-resistant/tolerant weeds has triggered the need to diversify the existing weed control practices [2,3]. Subsequently, there has been a growing interest in the utilization of natural products in the management of weeds. Secondary metabolites produced by plants have no direct role in the basic processes of plant growth and development. After being released into the environment, some of these bioactive molecules (allelochemicals) influence the growth and development of other surrounding species, a phenomenon known as allelopathy [4,5,6]. These compounds can improve a plant’s ability to compete in its local environment [7,8,9]. Allelochemicals interfere with various physiological processes of plants, including respiration, photosynthesis, and hormone balance, to affect the germination and growth of surrounding plants [10,11].

The phytotoxic effects of plant species have been explored to diversify existing weed management strategies for sustainable agriculture [12,13]. Plant extracts have been utilized to control pests [14], and isolated allelochemicals have the potential to be used in weed control or herbicide formulation [15]. However, allelopathy is a complex phenomenon since the production and release of plant secondary metabolites can be altered by environmental conditions. Seasonal changes in biotic and abiotic variables, such as pathogen presence [16], temperature [17], precipitation [18], and nutrient availability [19], can have a significant impact on the production and release of allelochemicals, which can contribute to seasonal fluctuations in plant phytotoxicity. Furthermore, soil bacteria can break down allelochemicals into less hazardous molecules or transform them into more toxic compounds [20,21]. Although the concentration of allelochemicals in plant tissues (flowers, leaves, stems, bark, and roots) might change during the growing season [22,23], most studies on the potential phytotoxicity of plants focus on a particular evaluation period during the season. However, understanding the potential phytotoxicity of plant species and gaining insight into the ecological interactions of plants with their environment necessitates the investigation of seasonal fluctuation [24].

*Rosmarinus officinalis* L. (Lamiaceae), also known as rosemary, is an evergreen shrub that grows wild in the Mediterranean region. A recent phylogenetic analysis merged the genus *Rosmarinus* with the genus *Salvia*. *Rosmarinus officinalis* is now known as *Salvia rosmarinus* [25,26,27]. Rosemary is an aromatic plant with needle-like leaves. The plant is now cultivated worldwide and has several reported therapeutic properties, including antidepressant [28], antiproliferative [29], and antidiabetic [30] activities. Diterpenes (such as carnosol and carnosic acid) and rosmarinic acid, both with strong antioxidant activity, have also been found in rosemary extracts [31,32,33]. The leaf extract of rosemary was reported to be potentially phytotoxic, and carnosic acid was reported as an allelochemical in the leaves of the plants [34]. Rosemary leaves also contain volatiles such as 1,8-cineole, which showed inhibitory effects on lettuce growth [35]. Carnosic acid has only been identified in a few plant species, all of which belong to the Lamiaceae family [36,37,38]. Richheimer et al. [39] reported carnosic acid concentrations in rosemary leaves between 1.7% and 3.9%. Subsequently, rosemary cultivars such as Daregal, VAU3, 4 English, Farinole, and Severn Seas were developed with higher levels of carnosic acid (4–10% on a weight basis of air-dried leaves) [40]. In addition, the concentration of carnosic acid in rosemary can also be modulated by growing conditions and the influence of genetic background. Climatic and environmental stress both affect the production of carnosic acid in rosemary [38], which further increases the importance of phytotoxic evaluation of the plant under Mediterranean climatic conditions. Although there are studies on the seasonal variation of carnosic acid concentration in rosemary, the seasonal variation in the biological activities of the plant has mainly focused on antioxidant activity [37,41].

Consequently, there is no available report on the relationship between carnosic acid concentration and the inhibitory activity of rosemary leaves. This study, therefore, aimed to investigate (i) how carnosic acid concentration in rosemary leaves changes with the season, (ii) which environmental factors play a role in this change, and (iii) whether this seasonal dependence of carnosic acid concentration is related to the inhibitory effect of leaves on lettuce seedling growth.

## 2. Materials and Methods

### 2.1. Collection of Plant Samples

**Figure 1:**
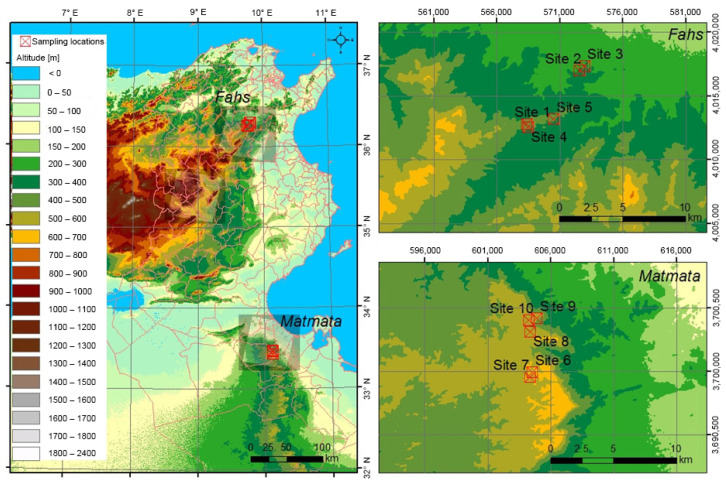
Map of sampling areas in Tunisia.

**Figure 2:**
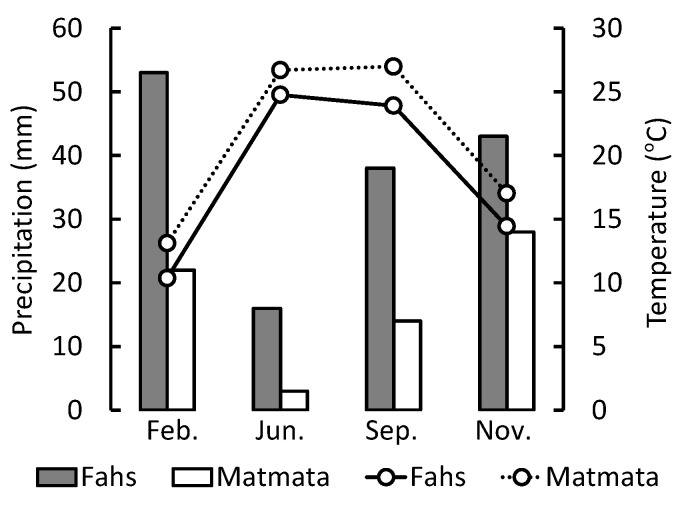
The monthly mean precipitation (bar graph: gray bar is Fahs; white bar is Matmata) and monthly mean temperature (line graph: the solid line is Fahs; the dotted line is Matmata) at the two sampling locations.

Plant samples were collected from the northern (Fahs) and southern (Matmata) parts of Tunisia (Figure 1). Matmata has an annual mean temperature of 20.6 °C, while Fahs has an annual mean temperature of 18.0 °C. The annual mean precipitation at Matmata and Fahs are 204 and 451 mm·year^−1^ respectively. Fahs belongs to the Mediterranean or steppe climate zone, while Matmata belongs to the desert climate zone with a drier climate [42]. The vegetations of the collection sites are affected by the Mediterranean climate, which has less precipitation in the summer. Matmata is drier than Fahs throughout the year. The monthly mean temperature and monthly mean precipitation at the sampling locations over the sampling period are shown in Figure 2.

The meteorological data for the sampling locations were assessed using WorldClim 2.1 [43]. These collecting sites were chosen because they feature rosemary-dominated vegetation, allowing rosemary plants from various climate zones to be compared. Sampling was done four times a year by randomly selecting five sites from each of the two areas of Fahs and Matmata. A total of 40 rosemary plant samples were collected from individual rosemary plants from June, September, and November of 2011, as well as February 2012. Sampling was done while avoiding spring when nutrients are used for flower growth rather than leaves. The sampling locations at Matmata were 535–620 m above sea level, whereas those at Fahs were 300–430 m above sea level. The elevation was recorded using a GPS (Colorado 300, Garmin, Olathe, KS, USA). The samples used in this study were only those collected in the growing season, each from a single rosemary plant (Table 1).

Each rosemary sample was given a unique ID (UT-ARENA management number) and stored at the Alliance for Research on the Mediterranean and North Africa’s herbarium at the University of Tsukuba in Japan. Rosemary leaves were collected from the tops of the individuals that were the most exposed to the sun. The collected samples were air-dried in a well-ventilated room and then placed in a light-shielding bottle for storage in a cool and dark place.

### 2.2. Extraction Procedure

The crude extracts were obtained from the air-dried rosemary leaf samples. In brief, 200 mg of air-dried rosemary leaves of each sample were accurately measured and placed into a 50 mL falcon tube containing 20 mL of solvent (80% ethanol). The leaf–solvent mixture was sonicated for 30 min at room temperature, filtered through filter paper No.1 (Advantec Toyo Roshi Kaisha, Tokyo, Japan), and centrifuged using Hitachi himac CR22N (6000 rpm, 10 min); then, the supernatants were collected. The residue was re-extracted using the same procedure as above, and the supernatants were combined and used as the working solutions.

### 2.3. Chemicals and Reagents

Carnosic acid used in this study was purchased from Tokyo Chemical Industry (TCI, Tokyo, Japan). Formic acid and acetonitrile for analytical chromatography were purchased from Fluka, Sigma-Aldrich (Steinheim, Germany) and Fisher Scientific (Madrid, Spain), respectively. A Milli-Q system from Millipore (Bedford, MA, USA) was used to purify the water used in all the analyses.

### 2.4. High-Performance Liquid Chromatography (HPLC) Analysis

A total of 50 mg of ground rosemary samples (leaves) was accurately weighed, put into a 50 mL falcon tube, and extracted, as described in the extraction procedure. An aliquot of the extract after centrifugation was filtered through a 0.2 μm syringe filter before the injection of 10 µL in LC-20AD liquid chromatography (Shimadzu, Japan) for the HPLC analysis. An Inertsil ODS 2 column (250 × 4.6 mm, 5 μm particles, GL Sciences Inc, Tokyo, Japan) was used. Mobile phases A and B were water with 0.1% formic acid and acetonitrile, respectively. The column temperature was kept at 30 °C, and the flow rate of the mobile phase was set at 0.5 mL·min^−1^. The following multistep gradient with different proportions of mobile phase B was applied: 0 min, 20% B; 10 min, 40% B; 15 min, 90% B maintained for 5 min. The initial conditions were maintained for 5 min. The analysis was monitored using an SPD-M20A detector at 210 nm. The quantification was done by comparing the peak areas of the targeted carnosic acid with the abundance of the compound in the corresponding standard used in the calibration curve. All chemical analyses were done in triplicate.

### 2.5. Phytotoxic Activity Bioassay

The radicle and hypocotyl elongation of *Lactuca sativa* (Great Lake 366, Takii Co., Kyoto, Japan) was evaluated in the phytotoxic activity bioassay using ethanol crude extracts of each of the 40 samples of rosemary leaves. In the phytotoxic activity bioassay, 40 samples of ethanol crude extracts of rosemary leaves were tested on the radicle and hypocotyl elongation of *Lactuca sativa*. The concentration range of the rosemary crude extracts (0.5, 1.0, 3.0, 5.0, and 10 mg DW·mL^−1^) was adapted from a previous study [34]. In a 27 mm diameter glass Petri dish, a filter paper (27 mm, Toyo Roshi Kaisha, Ltd., Tokyo, Japan) was inserted. A total of 0.7 mL of test solution was added to the filter paper and dried completely in vacuo. Five lettuce seedlings (pre-germinated for 20 h) were placed on the filter paper after adding 0.7 mL of 0.05% dimethyl sulfoxide (DMSO) and incubated (CN-25C, Mitsubishi Elec., Tokyo, Japan) for 52 h at 22 °C in dark conditions. The control treatments were set up with no crude extract but only 0.05% DMSO. Three replications were set for each treatment. The radicle and hypocotyl lengths were measured after the incubation period, and elongation percentages were calculated using the following equation:

(1) E=A/B×100

where *E* is the elongation percentage, *A* is the average length of radicle/hypocotyl in the treatment, and *B* is the average length of radicle/hypocotyl in the control.

### 2.6. Statistical Analysis

The IBM statistics tool SPSS (SPSS Inc., Chicago, IL, USA, version 21) was used to analyze the data. Data were subjected to a two-way analysis of variance (ANOVA) to determine the significant differences among the samples collected in different months and locations. The sampling months and locations were considered as the independent factors in the analysis. Mean differences among the treatments were compared using the Tukey test at *p* < 0.05. Pearson’s correlation analysis was conducted to establish significant relationships among the measured parameters.

## 3. Results

### 3.1. Variations in Carnosic Acid Concentration in Rosemary Leaves during the Growing Season

**Figure 3:**
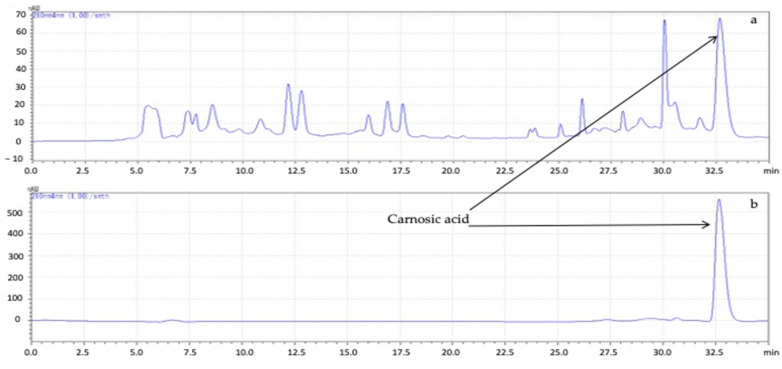
Chromatograph of an ethanol extract from rosemary leaves (**a**) and synthetic carnosic acid (**b**).

The concentration of carnosic acid in the leaves of rosemary samples collected from the two different locations (Fahs and Matmata) in Tunisia was studied over a growing season using reversed-phase high-performance liquid chromatography (RP-HPLC) (Figure 3). The equation for the calibration curve for carnosic acid was *y* = 84051*x* + 240721, *R*^2^ = 0.9994. The limit of detection (LOD) and limit of quantification (LOQ) were determined at signal-to-noise (S/N) ratios of 3 and 10, respectively. The LOD and LOQ were 0.0150 mg·g^−1^ and 0.0455 mg·g^−1^, respectively. This study focused primarily on carnosic acid, as it was previously found to be the major allelochemical responsible for the plant growth-inhibitory effect of rosemary leaves [34]. The results of this study showed that the accumulation of carnosic acid in rosemary leaves depended on the time of sampling.

**Figure 4:**
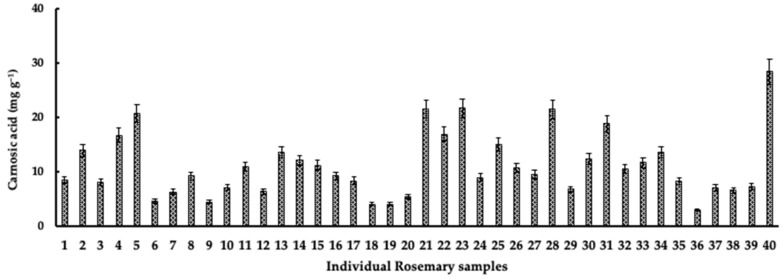
Distribution of carnosic acid concentration in the crude extracts of rosemary leaf samples collected from Tunisia (June 2011–February 2012). Values are the means of three replicates ± SD. CA: carnosic acid (expressed on a dry weight basis).

**Figure 5:**
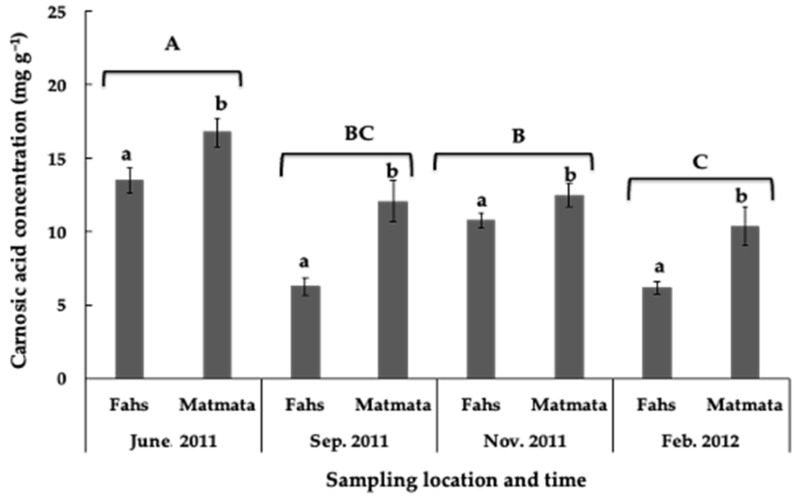
The effect of the sampling time and location on the concentration of carnosic acid in rosemary leaves. Data are expressed as mg·g^−1^ dry weight. Different letters (a, b, location for each month; A–C, sampling month during the season) above the error bars show treatments with significant differences throughout the season (*p* < 0.05). CA: carnosic acid.

The carnosic acid concentration in leaves of rosemary samples collected in Tunisia during the study period varied widely between 2.9 and 28.4 mg·g^−1^ dry weight (Figure 4). The results showed that the highest average carnosic acid concentration (15.1 mg·g^−1^ dry weight) was measured in June (early summer), while the lowest concentration was measured in February (8.3 mg·g^−1^ dry weight) (Figure 5). It was observed that the concentration of carnosic acid in the leaves of rosemary was higher in the samples from Matmata than in those from Fahs at all sampling times.

### 3.2. Influence of Precipitation, Elevation, and Temperature on Carnosic Acid Concentration in Rosemary Leaves

The two sampling locations had different annual precipitation, temperature, and elevation. Matmata has a hot climate, while Fahs has a moderately hot climate. To determine which environmental factors might be related to the observed seasonal variation in carnosic acid concentration, a Pearson correlation analysis was performed based on carnosic acid concentration and environmental factors (temperature, precipitation, and altitude) during sampling (Table 2). Carnosic acid concentration showed a significant positive correlation with temperature (*r* = 0.30; *p* < 0.05) and altitude (*r* = 0.33; *p* < 0.05). However, there was a significant inverse relationship between carnosic acid concentration and precipitation at the sampling locations (Table 2). The results show that temperature and precipitation variations influence the concentration of carnosic acid in rosemary leaves during the season.

### 3.3. Effects of Seasonality on the Plant Growth-Inhibitory Potential of Rosemary Leaves 

The concentration of carnosic acid in rosemary leaves showed seasonal variation and a significant relationship with precipitation and temperature. The study also investigated whether the seasonal variation in carnosic acid concentration could influence the phytotoxic activity of rosemary during the sampling season. The phytotoxic activity assay was tested on lettuce elongation. The inhibitory effect of rosemary leaf ethanol crude extracts on lettuce radicle and hypocotyl elongation was dose-dependent. The ranges of inhibition of lettuce radicle and hypocotyl elongation were 18.3–123% and 15.6–100% (percentage of control), respectively (Table S1). Lettuce hypocotyl elongation was more sensitive to rosemary crude extract than the radicle.

**Figure 6:**
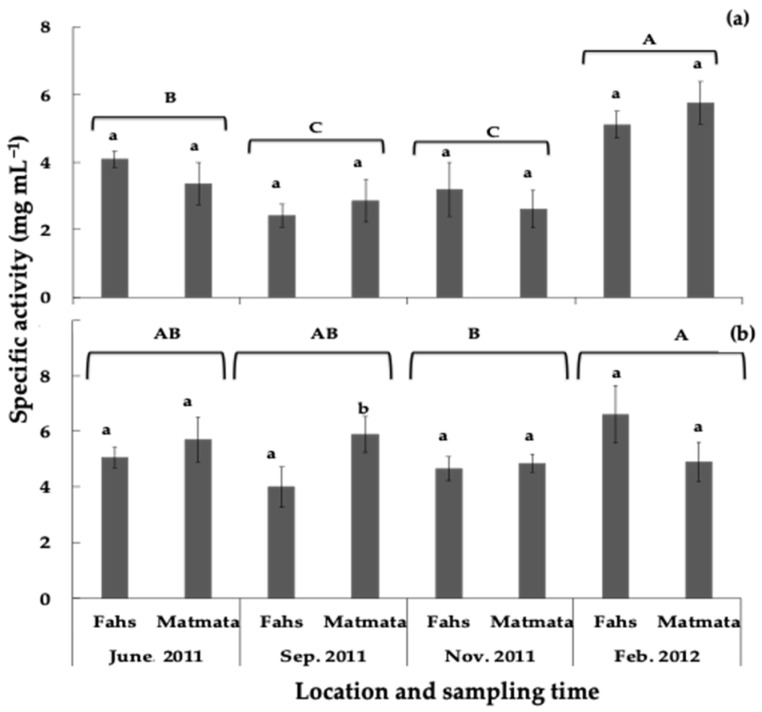
Effect of the sampling period and location on the growth-inhibitory activity of rosemary leaves on lettuce (**a**) hypocotyl and (**b**) radicle elongation. Different letters (a, b, location for each month; A–C, sampling month during the season) above the error bars show treatments with significant differences throughout the season (*p* < 0.05). Values are means ± SD (*n* = 5).

The concentration of rosemary leaf extracts required for 50% growth inhibition (EC_50_ or specific activity) of lettuce elongation was determined for all the collected rosemary samples. The inhibitory effect (expressed as EC_50_) on lettuce growth ranged from 2.1–8.6 mg DW·mL^−1^ and from 0.7–7.2 mg DW·mL^−1^ for radicle and hypocotyl, respectively (Table S1). The observed phytotoxicity of rosemary leaves on lettuce length growth showed seasonal variations. Samples collected in September and November had the lowest EC_50_ values (strong inhibition) for lettuce hypocotyl elongation (Figure 6a).

High EC_50_ values (low inhibition) for lettuce hypocotyl were measured in February at both locations, which coincided with the lowest carnosic acid concentration in rosemary leaves. The average specific activity of samples collected in November, September, June, and February on lettuce radicle elongation was 4.8, 5.0, 5.6, and 6.2 mg DW·mL^−1^, respectively (Figure 6b). Except for samples collected in September, there was no significant difference in inhibitory activity between sampling locations during the season (Figure 6). The effect of sampling location, sampling period, and their interaction on lettuce carnosic acid concentration and growth elongation are shown in Table 3. Except for the effect of sampling location on hypocotyl and radicle growth, all other effects and interactions were significant. However, the seasonal variation in phytotoxicity and concentration of carnosic acid in rosemary leaves should be evaluated over 1 year in a Mediterranean climate to fully understand this relationship.

### 3.4. Correlation between Carnosic Acid Concentration and Phytotoxicity of Rosemary Leaves

**Figure 7:**
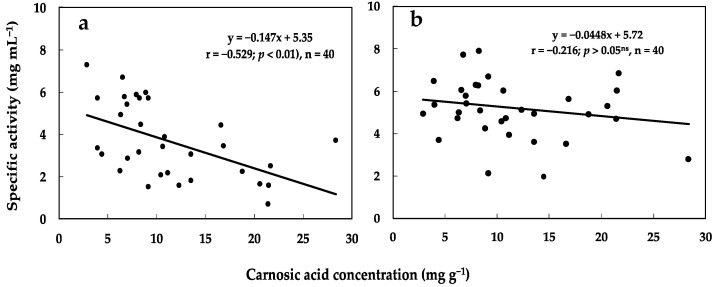
Relationship between carnosic acid concentration and phytotoxicity (expressed as EC_50_) of the leaf extract of rosemary on lettuce (**a**) hypocotyl and (**b**) radicle elongations.

To determine the relationship between carnosic acid concentration and phytotoxicity of rosemary leaf extracts, a Pearson correlation analysis was performed based on the results of carnosic acid concentration and EC_50_ (specific activity) of the leaves. The resulting graph represents a natural dose-response curve for carnosic acid in rosemary leaves. The correlation study showed a significant inverse relationship (*p* < 0.01; *r* = −0.529) between carnosic acid concentration and inhibitory effects (expressed as EC_50_ or specific activity) of rosemary leaves for hypocotyl elongation (Figure 7a). This result shows that the contribution of carnosic acid to the inhibitory effect of rosemary leaves on lettuce hypocotyl elongation is high, but low on radicle elongation. The results indicate that rosemary leaves with a high concentration of carnosic acid have great phytotoxic potential, which can be further explored. However, the degree of correlation between carnosic acid concentration and phytotoxicity of rosemary leaves indicates that other compounds may also contribute to the phytotoxicity of rosemary leaves.

## 4. Discussion

The RP-HPLC analysis of rosemary leaves collected from the two locations in Tunisia showed that carnosic acid concentration varied throughout the season (as shown in Figure 4). Other studies reported similar variations in carnosic acid concentration in rosemary samples from different geographical zones [44,45]. The average carnosic acid concentration was highest in early summer for both sampling locations in this study. In line with the results of this study, Hidalgo et al. [45] also reported an increase in carnosic acid concentration in rosemary leaves in summer (46.2 mg·g^−1^ in July 1996), while the lowest values were observed in February of the same year. In Brazil, the reported carnosic acid concentration in rosemary leaves was highest in leaf samples collected in summer [46]. In contrast, Luis and Johnson [37] observed a decrease in the carnosic acid concentration of about 50% during the summer months characterized by high temperatures. The discrepancy in the concentration of carnosic acid in rosemary leaves could be due to the influence of growing conditions and other factors. The influence of environmental factors on the variation of carnosic acid concentration in rosemary leaves was reported previously [46,47]. The seasonal variations in carnosic acid concentration observed in this study may indicate that the synthesis of the compound is influenced by changes in certain climatic factors.

The results also showed a relationship between environmental conditions at the time of sampling and carnosic acid concentration in rosemary leaves. Temperature, precipitation, and elevation of sampling locations showed significant correlations with carnosic acid concentration in rosemary leaves. Similar to the results of this study, Hidalgo et al. [45] reported increasing carnosic acid concentration in rosemary leaves with increasing temperature. Lemos et al. [46] also reported the highest carnosic acid concentration in the month with the highest temperature. In contrast, Munne-Bosch et al. [48] reported a negative linear relationship between carnosic acid concentration and temperature. However, an increased amount of carnosic acid was detected during the summer with high rainfall and temperature in Brazil [46]. Borras et al. [44] reported that the observed variations in the altitude of sampling locations had significant effects on the concentration of plant metabolites (including carnosic acid) in rosemary leaves. Compared to other native Mediterranean plants, rosemary can withstand prolonged drought by avoiding damage to its photosynthetic organs [47]. Seasonal variation is associated with certain changes in soil moisture and temperature, which may lead to variations in the biosynthetic pathways of primary and secondary metabolites [17,18]. Carnosic acid was found mainly in June 2011, followed by September 2011 and November 2011. The biosynthetic pathway of terpenes could explain this observation. Terpenes are synthesized in the cytosol and plant plastids [49]. The pathway leads to the formation of sesquiterpenoids in the cytoplasm and the formation of diterpenes and tetraterpenes in the plastid. However, these processes are associated with the capture of sunlight and a photoprotective function in cell membranes [49]. Thus, according to the biosynthetic mechanisms, rosemary leaves harvested in June 2011 increased the synthesis of terpenes (including carnosic acid) in plastids at the high temperatures (26.6 °C). Moreover, carnosic acid is one of the most important antioxidants in rosemary leaves, and its concentration increases under stress conditions [46]. It should be considered that the production of carnosic acid in rosemary depends on the genetic background, plant part, and growing conditions [50], which could also explain part of the discrepancy between the results reported in different studies.

Although carnosic acid was reported as the principal allelochemical in rosemary leaves [34], other compounds found in the plant, such as ferulic, caffeic, gallic, chlorogenic, and rosmarinic acids, have been linked to phytotoxicity [51]. The antioxidative mechanism of carnosic acid in plants has been reported [52]; however, there has been no reported study on its mode of action as a plant growth inhibitor. Since other compounds contribute to the inhibitory effects of rosemary leaves, the physiological actions of some of these compounds are discussed. According to Araniti et al. [53], rosmarinic acid inhibited the main reactive oxygen species (ROS)-scavenging enzymes, resulting in high ROS levels that cause alterations in mitochondrial ultrastructure and function, leading to cell death in *Arabidopsis* seedlings. Rudrappa et al. [54] asserted that gallic acid elevated the level of ROS in the roots of *Arabidopsis*. The activated ROS caused the root architecture of susceptible plants to be disrupted by impairing the microtubule assembly. According to dos Santos et al. [55], ferulic acid may be channelled into the phenylpropanoid pathway, where it may increase the quantity of lignin monomer in the cell wall, hardening the cell wall and inhibiting root growth. Similarly, caffeic acid channelled into the phenylpropanoid pathway increased lignin monomers that solidify the cell wall and inhibit root growth [56]. 1,8-Cineole, a significant essential oil in rosemary, decreased root growth in other plants by impeding DNA synthesis in the apical meristem of *Brassica campestris* roots [57]. Monoterpenes, which are abundant in rosemary, inhibited chlorophyll content, as well as the biosynthesis of several phenolic compounds [58].

The rosemary leaves sampled in this study showed variations in carnosic acid concentration, suggesting that the growth-inhibitory effect of the leaves may change over the season. The study further confirmed that the phytotoxicity of rosemary leaves changed during the sampling period. The changes in carnosic acid concentration and the expression of biological activities during different seasons have been reported in other studies [46,59,60]. Although the antimicrobial activity of rosemary leaves changed during the growing season [61], seasonal changes in the phytotoxicity of rosemary have not been reported. The concentration of carnosic acid in rosemary leaves showed a significant correlation (*r* = −0.529; *p* < 0.01) with growth inhibition at the hypocotyl of lettuce. Our results agree with other studies that showed that allelochemicals and growth inhibition are related in allelopathic species. Ben-Hammaouda et al. [62] reported that the phytotoxicity of sorghum hybrids had a positive correlation (*r* = 0.66) with the total concentration of phenolic compounds. Similarly, Reberg-Horton et al. [63] reported that the inhibitory effect of aqueous extracts of *Secale cereale* tissue correlated with the amount of DIMBOA extracted from the harvested tissue. In another study, the concentration of phenolic acids together with DIBOA and DIMBOA explained about 90% of the variation in growth inhibition observed in annual ryegrass [64]. Although a significant relationship was found between carnosic acid concentration and growth inhibition, the contribution of other compounds to rosemary leaf phytotoxicity should not be ignored.

## 5. Conclusions

The concentration of carnosic acid in rosemary leaves and the inhibitory effect of ethanolic extracts of rosemary leaves were both influenced by seasonal variations. The carnosic acid concentration in rosemary leaves peaked in early summer at both sampling locations in Tunisia and then gradually decreased until winter. Rosemary leaf phytotoxicity (expressed as EC_50_) followed a similar pattern throughout the season and showed a significant (inverse) relationship with carnosic acid content. It is important to evaluate the seasonal variation in the inhibitory activity of rosemary leaves to avoid over-or underestimating the phytotoxicity of the plant. The efficacy of rosemary as a potential weed control agent needs further investigation under field conditions.

## Figures and Tables

**Table 1:** Description of rosemary sampling sites (dates, areas, elevation) and specimen codes.

No.	Collection Date	Geographical Area	Elevation (m)	Sample Codes
1	June 2011	Fahs	430	UT-ARENA 00327
2	June 2011	Fahs	320	UT-ARENA 00334
3	June 2011	Fahs	300	UT-ARENA 00340
4	June 2011	Fahs	430	UT-ARENA 00349
5	June 2011	Fahs	420	UT-ARENA 00357
6	June 2011	Matmata	620	UT-ARENA 00364
7	June 2011	Matmata	585	UT-ARENA 00371
8	June 2011	Matmata	575	UT-ARENA 00379
9	June 2011	Matmata	535	UT-ARENA 00387
10	June 2011	Matmata	555	UT-ARENA 00395
11	September 2011	Fahs	430	UT-ARENA 00402
12	September 2011	Fahs	320	UT-ARENA 00411
13	September 2011	Fahs	300	UT-ARENA 00417
14	September 2011	Fahs	430	UT-ARENA 00426
15	September 2011	Fahs	420	UT-ARENA 00434
16	September 2011	Matmata	620	UT-ARENA 00442
17	September 2011	Matmata	585	UT-ARENA 00453
18	September 2011	Matmata	575	UT-ARENA 00460
19	September 2011	Matmata	535	UT-ARENA 00469
20	September 2011	Matmata	555	UT-ARENA 00478
21	November 2011	Fahs	430	UT-ARENA 00515
22	November 2011	Fahs	320	UT-ARENA 00523
23	November 2011	Fahs	300	UT-ARENA 00535
24	November 2011	Fahs	430	UT-ARENA 00543
25	November 2011	Fahs	420	UT-ARENA 00550
26	November 2011	Matmata	620	UT-ARENA 00559
27	November 2011	Matmata	585	UT-ARENA 00565
28	November 2011	Matmata	575	UT-ARENA 00574
29	November 2011	Matmata	535	UT-ARENA 00583
30	November 2011	Matmata	555	UT-ARENA 00587
31	February 2012	Fahs	430	UT-ARENA 00617
32	February 2012	Fahs	320	UT-ARENA 00622
33	February 2012	Fahs	300	UT-ARENA 00628
34	February 2012	Fahs	430	UT-ARENA 00633
35	February 2012	Fahs	420	UT-ARENA 00638
36	February 2012	Matmata	620	UT-ARENA 00647
37	February 2012	Matmata	585	UT-ARENA 00652
38	February 2012	Matmata	575	UT-ARENA 00657
39	February 2012	Matmata	535	UT-ARENA 00662
40	February 2012	Matmata	555	UT-ARENA 00667

UT-ARENA: the University of Tsukuba Alliance for Research on the Mediterranean and North Africa.

**Table 2:** Pearson correlation analysis for carnosic acid concentration, precipitation, elevation, and temperature.

Attribute	Elevation	Precipitation	Temperature	CA Concentration
Elevation	1.00			
Precipitation	−0.61 **	1.00		
Temperature	0.19	−0.71 **	1.00	
CA amt	0.33 *	−0.49 *	0.30 *	1.00

CA: carnosic acid. Significance level: * *p* < 0.05, ** *p* < 0.01.

**Table 3:** Summary of the analysis of variance (ANOVA) for carnosic acid concentration, growth elongations, sampling location, and period.

Source of Variation	DF	CA Concentration		Hypocotyl Growth		Radicle Growth	
		MS	*p*-level	MS	*p*-level	MS	*p*-level
Location	1	167.4	<0.001 **	0.02	>0.05	0.52	>0.05
Month	3	61.9	<0.001 **	12.7	<0.001 **	1.6	<0.05 *
Location × Month	3	8.9	<0.05 *	0.9	<0.05 *	4.5	<0.001 **
Error	24	2.2		0.3		0.5	
Total	31						
R^2^		0.88		0.85		0.63	

* Significant at the 0.05 level of probability. ** Significant at the 0.01 level of probability. *p* > 0.05: not significant. CA: Carnosic acid. Growth is expressed as a percentage of the control. MS: means of squares.

## Data Availability

Data included in the article with Supplementary Materials.

## Supplementary Materials

The following supporting information can be downloaded at https://www.mdpi.com/article/10.3390/plants11050673/s1: Table S1. The effects of rosemary leaf samples from different seasons and at different locations on lettuce growth elongation; Table S2. Moran’s I statistic.
