# Supplementary material for: Seasonal Changes in the Plant Growth-Inhibitory Effects of Rosemary Leaves on Lettuce Seedlings
Source: Plants (Basel). 2022 Mar 1;11(5):673. doi: 10.3390/plants11050673 (PMC8912698; doi:10.3390/plants11050673)
Supplement: Supplementary file 1 [file plants-11-00673-s001.zip › plants-1598747-supplementary.pdf]

plants

MDPI

Article

Seasonal Changes in the Plant Growth-Inhibitory Effects of Rosemary Leaves on Lettuce Seedlings

Kwame Sarpong Appiah [1,2,*], Richard Ansong Omari [3,4], Siaw Onwona-Agyeman [5], Christiana Adukwei Amoatey [2], John Ofosu-Anim [6], Abderrazak Smaoui [7], Abdelkarim Ben Arfa [8], Yoko Suzuki [9], Yosei Oikawa [1], Shin Okazaki [1], Keisuke Katsura [1], Hiroko Isoda [10], Kiyokazu Kawada [10,*] and Yoshiharu Fujii [1]

1 Department of International Innovative Agricultural Science, Tokyo University of Agriculture and Technology, 3-5-8 Saiwaicho, Fuchu, Tokyo 183-8509, Japan; yosei@cc.tuat.ac.jp (Y.O.); sokazaki@cc.tuat.ac.jp (S.O.); kkatsura@go.tuat.ac.jp (K.K.); yfujii@cc.tuat.ac.jp (Y.F.)
2 Department of Crop Science, College of Basic and Applied Science, University of Ghana, Legon, Accra P.O. Box LG 44, Ghana; camoatey@ug.edu.gh
3 Leibniz Centre for Agricultural Landscape Research, Institute of Land Use Systems, Eberswalder Str. 84, 15374 Muencheberg, Germany; talk2jafakingonline@gmail.com
4 Institute of Agriculture and Horticulture, Faculty of Life Science, Humboldt-University of Berlin, Albrecht-Thaer-Weg 5, 14195 Berlin, Germany
5 Institute of Agriculture, Tokyo University of Agriculture and Technology, 3-5-8 Saiwaicho, Fuchu, Tokyo 183-8509, Japan; agyeman@cc.tuat.ac.jp
6 School of Architecture and Science, Central University, Tema P.O. Box 2305, Ghana; jofosuanim@gmail.com
7 Centre of Biotechnology of Borj Cédria, BP 901 Hammam-Lif, Borj Cedria 2025, Tunisia; abderrazak.smaoui@gmail.com
8 L'Institut des Régions Arides, Route du Djorf Km 22.5, Médenine 4119, Tunisia; abdelkarim.benarfa@ira.rnrt.tn
9 Aromatic Repos, AHOLA, A2 Soleil Jiyugaoka, 1-21-3, Jiyugaoka, Meguro, Tokyo 152-0035, Japan; yoko86252539@gmail.com
10 School of Life and Environmental Sciences, University of Tsukuba, Tennoudai 1-1-1, Tsukuba, Ibaraki 305-8572, Japan; isoda.hiroko.ga@u.tsukuba.ac.jp
* Correspondence: ksappiah90@gmail.com (K.S.A.); kawada.kiyokazu.gu@u.tsukuba.ac.jp (K.K.)

.

Citation: Appiah, K.S.; Omari, R.A.; Onwona-Agyeman, S.; Amoatey, C.A.; Ofosu-Anim, J.; Smaoui, A.; Arfa, A.B.; Suzuki, Y.; Oikawa, Y.; Okazaki, S.; et al. Seasonal Changes in the Plant Growth-Inhibitory Effects of Rosemary Leaves on Lettuce Seedlings. *Plants* **2022**, *11*, 673. https://doi.org/10.3390/plants11050673

Academic Editors: Sofia Rhizopoulou, Maria Karatassiou and Efi Levizou

Received: 30 January 2022
Accepted: 24 February 2022
Published: 1 March 2022

Publisher's Note: MDPI stays neutral with regard to jurisdictional claims in published maps and institutional affiliations.

Copyright: © 2022 by the authors. Licensee MDPI, Basel, Switzerland. This article is an open access article distributed under the terms and conditions of the Creative Commons Attribution (CC BY) license (https://creativecommons.org/licenses/by/4.0/).

Table S1. The effects of rosemary leaves collected at different seasons and locations on lettuce growth elongation.

Sample period (month, year)	Location	Sample Codes	Growth elongation (% of control) at different concentrations											
			0.5 mg mL^{-1}		1.0 mg mL^{-1}		3.0 mg mL^{-1}		5.0 mg mL^{-1}		10 mg mL^{-1}		EC_{50} mg mL^{-1}	
			R	H	R	H	R	H	R	H	R	H	R	H
June 2011	Fahs	UT-ARENA 00327	109.7	86.0	80.0	51.4	43.8	45.8	39.3	45.8	23.8	31.8	5.1	4.4
		UT-ARENA 00334	118.8	79.5	103.4	48.4	71.7	44.3	47.8	38.5	30.4	30.3	6.6	3.6
		UT-ARENA 00340	120.5	100.7	116.3	67.9	61.1	48.1	41.0	45.3	29.3	36.8	6.3	5.8
		UT-ARENA 00349	80.3	58.0	67.0	53.0	39.0	41.0	31.4	37.0	22.0	33.0	3.5	1.6
		UT-ARENA 00357	115.4	94.1	96.6	46.6	41.9	47.5	32.2	42.4	24.5	31.4	5.3	4.4
	Matmata	UT-ARENA 00364	119.7	80.5	100.8	65.5	57.1	58.6	39.8	49.4	28.6	36.8	6.0	6.0
		UT-ARENA 00371	106.5	52.5	91.5	45.9	55.1	40.2	38.4	39.3	26.5	32.0	5.7	3.5
		UT-ARENA 00379	116.9	61.5	86.4	48.4	67.6	45.9	51.5	42.6	36.0	37.7	6.9	2.5
		UT-ARENA 00387	86.9	69.5	72.9	43.5	44.9	37.4	31.2	35.1	25.2	28.2	4.3	1.6
		UT-ARENA 00395	120.2	82.7	118.1	58.6	57.1	42.1	33.0	36.1	26.6	31.6	5.9	4.0
September 2011	Fahs	UT-ARENA 00402	76.4	48.9	57.5	43.8	28.5	34.3	22.5	30.7	14.5	22.6	2.1	1.5
		UT-ARENA 00411	102.6	82.5	94.7	58.3	36.8	24.7	28.2	22.4	22.5	20.2	4.7	2.3
		UT-ARENA 00417	104.1	81.1	80.4	43.3	46.5	37.8	37.3	37.8	32.1	32.3	5.4	2.8
		UT-ARENA 00426	96.8	82.3	61.8	48.5	39.2	37.7	28.8	36.2	16.5	26.9	3.7	3.0
		UT-ARENA 00434	93.6	71.3	66.1	52.1	45.0	47.9	29.9	37.2	18.7	33.0	4.0	3.4
	Matmata	UT-ARENA 00442	105.7	72.0	96.8	60.6	53.7	40.2	50.5	40.2	26.3	27.3	6.0	3.4
		UT-ARENA 00453	105.9	59.8	77.1	43.9	39.9	40.9	33.3	31.8	23.5	26.5	4.7	0.7
		UT-ARENA 00460	103.6	61.4	92.4	38.6	45.5	39.4	40.0	27.3	20.4	21.2	5.1	1.6
		UT-ARENA 00469	114.4	93.3	106.7	65.0	82.0	50.0	46.5	40.0	44.7	41.7	7.7	5.8
		UT-ARENA 00478	97.3	82.4	90.0	49.6	45.5	42.9	45.2	42.0	36.9	28.6	6.1	3.7
November 2011	Fahs	UT-ARENA 00515	107.2	66.9	86.5	41.2	41.8	34.6	29.9	31.6	20.4	22.8	4.7	3.9
		UT-ARENA 00523	93.6	77.3	77.6	46.2	33.9	36.4	25.8	28.8	19.7	25.0	4.0	2.2
		UT-ARENA 00535	108.6	56.1	78.7	38.6	42.5	34.1	35.5	31.1	23.9	28.0	4.9	1.8
		UT-ARENA 00543	106.4	50.8	92.1	47.5	45.3	42.4	37.1	37.3	19.5	29.7	5.0	3.7
		UT-ARENA 00550	107.8	97.2	96.0	64.6	46.2	51.0	28.2	38.5	20.9	28.1	5.0	4.9
	Matmata	UT-ARENA 00559	95.5	48.9	79.8	38.5	41.4	39.3	40.2	43.7	24.2	25.9	4.9	2.2
		UT-ARENA 00565	94.8	54.1	77.6	55.9	49.7	44.1	35.7	41.4	17.5	26.1	4.6	2.1
		UT-ARENA 00574	106.4	62.4	69.6	40.6	41.9	36.1	33.7	32.3	21.1	26.3	4.5	3.9
		UT-ARENA 00583	90.9	53.7	62.5	33.6	36.9	32.9	28.0	26.8	21.6	23.5	3.6	3.0
		UT-ARENA 00587	122.1	75.7	105.2	50.4	50.6	45.2	44.2	37.4	32.9	28.7	6.3	3.2
February 2012	Fahs	UT-ARENA 00617	123.4	98.6	87.6	75.6	59.3	48.3	38.0	32.3	28.3	27.9	6.7	5.7
		UT-ARENA 00622	115.2	98.6	92.3	62.3	55.3	42.3	31.1	31.8	25.3	26.6	7.9	5.7
		UT-ARENA 00628	110.1	71.8	94.0	55.5	47.4	44.5	35.1	39.1	25.0	28.2	5.4	3.4
		UT-ARENA 00633	110.2	88.9	85.3	60.3	58.3	49.3	24.7	29.9	39.3	26.3	6.5	5.7
		UT-ARENA 00638	112.3	95.3	96.3	68.6	58.3	45.6	26.2	28.9	32.9	30.6	8.6	5.9
	Matmata	UT-ARENA 00647	110.1	58.2	85.9	40.4	40.3	39.7	36.9	31.9	20.5	25.5	5.0	7.3
		UT-ARENA 00652	101.4	72.9	81.9	58.3	61.2	53.1	40.2	45.8	29.7	40.6	5.8	5.4
		UT-ARENA 00657	117.1	78.6	100.6	70.2	55.1	58.3	42.0	53.6	31.0	40.5	6.1	6.7
		UT-ARENA 00662	111.4	79.0	102.2	59.0	59.0	46.7	42.4	42.9	28.8	34.3	6.9	4.5
		UT-ARENA 00667	118.2	98.3	80.3	62.6	60.3	45.6	37.2	30.8	18.3	15.6	2.8	3.7

R: Radicle % of control, H: Hypocotyl % of control, EC_{50}: Effective concentration to cause half-maximal inhibition.

Table S2: Moran's I Statistic

Variable	Moran's I	Expected (I)	sd	*p*-value
Temperature	-0.043124	-0.14286	0.184716	0.5892478
Precipitation	-0.459758	-0.14286	0.176211	0.0721109
CA amount	-0.036888	-0.14286	0.167392	0.5266963
EC_{50} (Radicle)	-0.142857	-0.14286	0.156981	0.1862481
EC_{50} (Hypocotyl)	-0.62431	-0.14286	0.171675	0.0050405

p- value > 0.05 means absence of spatial autocorrelation

Null Hypothesis: Spatial Randomization/Zero spatial autocorrelation
